# The intravenous and oral pharmacokinetics of lotilaner in dogs

**DOI:** 10.1186/s13071-017-2475-z

**Published:** 2017-11-01

**Authors:** Céline E. Toutain, Wolfgang Seewald, Martin Jung

**Affiliations:** Elanco Animal Health Inc., Mattenstrasse 24a, CH-4058 Basel, Switzerland

**Keywords:** Lotilaner, Isoxazoline, Pharmacokinetics, Dog, Oral, Intravenous, Food effect, Fed, Fasted

## Abstract

**Background:**

Lotilaner is a new oral ectoparasiticide from the isoxazoline class developed for the treatment of flea and tick infestations in dogs. It is formulated as pure *S*-enantiomer in flavoured chewable tablets (Credelio™). The pharmacokinetics of lotilaner were thoroughly determined after intravenous and oral administration and under different feeding regimens in dogs.

**Methods:**

Twenty-six adult beagle dogs were enrolled in a pharmacokinetic study evaluating either intravenous or oral administration of lotilaner. Following the oral administration of 20 mg/kg, under fed or fasted conditions, or intravenous administration of 3 mg/kg, blood samples were collected up to 35 days after treatment. The effects of timing of offering food and the amount of food consumed prior or after dosing on bioavailability were assessed in a separate study in 25 adult dogs. Lotilaner blood concentrations were measured using a validated liquid chromatography/tandem mass spectrometry (LC-MS/MS) method. Pharmacokinetic parameters were calculated by non-compartmental analysis. In addition, in vivo enantiomer stability was evaluated in an analytical study.

**Results:**

Following oral administration in fed animals, lotilaner was readily absorbed and peak blood concentrations reached within 2 hours. The terminal half-life was 30.7 days. Food enhanced the absorption, providing an oral bioavailability above 80% and reduced the inter-individual variability. Moreover, the time of feeding with respect to dosing (fed 30 min prior, fed at dosing or fed 30 min post-dosing) or the reduction of the food ration to one-third of the normal daily ration did not impact bioavailability. Following intravenous administration, lotilaner had a low clearance of 0.18 l/kg/day, large volumes of distribution V_z_ and V_ss_ of 6.35 and 6.45 l/kg, respectively and a terminal half-life of 24.6 days. In addition, there was no in vivo racemization of lotilaner.

**Conclusions:**

The pharmacokinetic properties of lotilaner administered orally as a flavoured chewable tablet (Credelio™) were studied in detail. With a T_max_ of 2 h and a terminal half-life of 30.7 days under fed conditions, lotilaner provides a rapid onset of flea and tick killing activity with consistent and sustained efficacy for at least 1 month.

**Electronic supplementary material:**

The online version of this article (10.1186/s13071-017-2475-z) contains supplementary material, which is available to authorized users.

## Background

Infestation with fleas and ticks is an indisputable health challenge for dogs throughout the world as they can cause a variety of dermatological conditions and anaemia. Female fleas begin laying eggs within 24–36 h of acquiring a host, can produce as many as 40 to 50 eggs per day [1], rapidly creating an infestation. While the majority of 3-host ticks infesting dogs are acquired from the external environment and require additional hosts, *Rhipicephalus sanguineus* (*sensu lato*) (*s*.*l*.) prefers to feed on dogs during larval, nymphal and adult stages and will infest homes, kennels and veterinary hospitals. The *R. sanguineus* (*s*.*l*.) life-cycle can be completed entirely indoors in as little as 2 months under ideal conditions [2]. Additionally, fleas and ticks are competent vectors for numerous infectious diseases and secondary endoparasitic infections in dogs and vector-borne disease agents transmissible to people and other animal species [3]. Anecdotal reports of ectoparasite populations with reduced sensitivity to older flea and tick products are increasing [4]. Based on these issues, it is important to find new ways to rapidly address both fleas and ticks infestations.

**Fig. 1:**
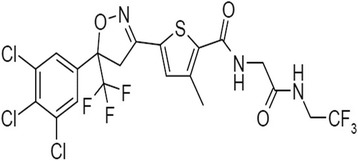
Molecular structure of lotilaner ((S)-5-[5-(3,4,5-trichloro-phenyl)-5-trifluoromethyl-4,5-dihydroisoxazol-3-yl] -3-methyl-thiophene-2-carboxylic acid [(2,2,2-trifluoro-ethylcarbamoyl)-methyl]-amide)

The isoxazolines are a novel family of compounds shown to have activity against fleas and ticks [5–7]. Compounds from a library of over 1000 structures were screened against insects and acari in in vitro and in rodent studies. The lead candidate lotilaner, i.e. (*S*)-5-[5-(3,4,5-trichloro-phenyl)-5-trifluoromethyl-4,5-dihydroisoxazol-3-yl]-3-methyl-thiophene-2-carboxylic acid [(2,2,2-trifluoro-ethylcarbamoyl)-methyl]-amide (Fig. 1) emerged as the most appropriate candidate when screened for both efficacy and safety. It has a molecular weight of 596.76, a measured log Pow (octanol/water partition coefficient) of 5.3. Lotilaner, as sarolaner [6] or afoxolaner [8], is highly bound to dog plasma proteins (unpublished data). With a chiral centre, *R*- and *S*- enantiomers can exist, the *S*- enantiomer being lotilaner. As a difference in potency of 10–100 fold between lotilaner and its opposite enantiomer (*R*-enantiomer) was measured in vitro against *Ctenocephalides felis* and *R. sanguineus* (*s.l.*) (unpublished data), Credelio™ was developed as the pure *S*-enantiomer. This reduces the amount of active ingredient in the final formulation, eliminates the need for the inactive compound to be metabolised and/or excreted by the patient and limits the possibility of secondary pharmacodynamic effects. In other words, the same dose, if administered as pure *S*-enantiomer, provides better efficacy and longer protection period as compared to the racemate, provided that there is no in vivo racemization.

Isoxazolines are potent inhibitors of γ-aminobutyric acid (GABA)-gated chloride channels (GABACls) [9, 10]. The GABA-mediated chloride influx leads to hyperpolarization of the cellular membrane and generates an inhibitory postsynaptic potential, which decreases the probability of an action potential. Insects and other invertebrates possess GABACls that are expressed not only in the central nervous system, where they generate inhibitory potentials for the correct integration of neuronal signals but also at peripheral neuromuscular sites, where they promote muscular relaxation. In the presence of lotilaner, GABACls are not able to open upon GABA stimulation, defining this molecule as an antagonist of GABACls. Parasites exposed to lotilaner endure a spastic paralysis leading to their starvation and death (unpublished data). In addition, isoxazolines are proven to be specific to insect and acari neuroreceptors, rather than mammalian neuroreceptors. The lack of effect on the mammalian nervous system at clinically relevant doses was confirmed in numerous laboratory and target animal safety studies. Repeated elevated doses administered orally at four-week intervals to young dogs at 8 weeks of age demonstrated that at the minimum dose rate of 43 mg/kg/month lotilaner has a wide margin of safety [11].

At a dose of 20 mg/kg, lotilaner has been shown to provide a rapid onset of activity against fleas and ticks that are sustained for at least 1 month following treatment [12–16]. Lotilaner is formulated as flavoured chewable tablets (Credelio™) and is to be administered orally on a monthly basis. To provide insights into the properties of lotilaner, support dose determination and formulation development, studies were performed in adult dogs to determine the pharmacokinetic profile after intravenous and oral administration, and to describe the effect of feeding on pharmacokinetic parameters.

## Methods

### Animal management

Purpose-bred mixed sex adult Beagle dogs and weighing approximately 9 to 16 kg were used. Each animal was uniquely identified and acclimatized to the study conditions for at least 1 week. Only healthy animals were included and suitability was evaluated by physical examination and clinical pathology. Dogs were housed indoors, in climate-controlled facilities in accordance with accepted laboratory animal care and use guidelines. They were kept in small groups except for the days around treatment administration where dogs were housed individually for at least 1 day, to avoid potential cross contamination between animals. Dogs were allowed the daily opportunity for outdoor exercise and social interaction. They were fed once daily with an appropriate ration of a commercial canine feed and water was available *ad libitum*. Dogs were observed for general health, behaviour and appetite at least once daily throughout the studies. All animals returned to their normal housing facilities on completion of the studies.

### Experimental designs

#### Study 1

In the first study, designed to investigate the effect of feeding on lotilaner pharmacokinetics after oral administration, 25 mixed sex adult dogs were allocated to five treatment groups with five dogs in each group. Each dog received a single oral administration of a close-to-final tablet formulation, at the target dose of 15 mg/kg lotilaner (the initially intended therapeutic dose). Dogs were fasted overnight and five different feeding regimes were tested as follows: dogs received their full daily food allowance (i) 30 min prior to; (ii) at the same time; (iii) 30 min after; (iv) 5 h after treatment administration; or (v) only one-third of their full daily food allowance at the same time of treatment administration. Blood specimens were collected from the jugular vein in K3-EDTA tubes at pre-dose and at 30 min, at 1, 2, 4, 8, 24, 48 and 72 h and at 7, 14 and 21 days post-treatment.

#### Study 2

In the second study, intended to determine the pharmacokinetic profile of lotilaner after intravenous and oral administration, 26 mixed sex adult dogs were allocated to three treatment groups as follows: one intravenous group of eight dogs, one oral group of 12 dogs treated 30 (± 5) min after feeding, and one oral fasted group of six dogs. Each dog from the oral groups received a single administration of the final tablet formulation (containing 35% *w*/*w* lotilaner), at the target minimum dose of 20 mg/kg lotilaner, which was the final therapeutic dose. Each dog from the intravenous group received a single administration of lotilaner in a solution consisting of 23% *w*/*v* physiological saline and tetraglycol ad 100% *w*/*v*, at the target dose of 3 mg/kg lotilaner, which was a dose known to be well tolerated intravenously. Blood specimens were collected from the jugular vein in K3-EDTA tubes at pre-dose and at 5 min (intravenous only), 30 min, at 1, 2, 4, 8, 24, 48 and 72 h and 7, 14, 21, 28 and 35 days post-treatment.

For both studies, whole blood specimens were stored frozen (for a maximum of 5 months) at approximately −20 °C until analysis with a validated LC-MS/MS method. During validation, stability at storage conditions was demonstrated for at least 9 months.

### Analysis of lotilaner in blood

Lotilaner was quantitatively analysed in blood using an analytical method involving liquid chromatography with tandem mass spectrometry detection (LC-MS/MS). Whole dog blood samples (80 μl) were extracted by precipitation with acetonitrile and further diluted with acetonitrile. A proprietary closely related chemical analogue was used as the internal standard. Ten microliters of each diluted supernatant were chromatographed by HPLC on a reversed-phase column [Thermo Betasil C18, 5 μm (50 × 4.6 mm)] with an isocratic mobile phase consisting of 0.1% formic acid and acetonitrile (15:85 *v*:*v*) using a flow rate of 0.8 ml/min and quantitatively analyzed on an AB Sciex API 5000 or API 5500 triple quadrupole mass spectrometer system using the negative Turbo IonSpray ionization mode and multiple reaction monitoring (MRM) of the transition m/z 596 to 181 for lotilaner.

The method was validated over a linear range of 6.8 to 6800 ng/ml, with a lower limit of quantification (LLOQ) of 6.8 ng/ml, according to FDA and EMA guidelines [17, 18]. Mean inter-day precision was 14.9% at LLOQ and ranged between 3.4 and 7.8% at the other levels and the mean inter-day accuracy ranged between 100.3 and 103.6%. In addition, specificity, dilution integrity, recovery and matrix effect, carryover, and stability in matrix and solutions were established. Long-term stability in frozen blood at −20 °C was demonstrated over 9 months.

### Enantiomeric stability

The in vivo enantiomeric stability of lotilaner was investigated in an analytical study. Blood specimens from 16 adult dogs which had received a single oral administration of the pure enantiomer drug at 15 mg/kg (tablet or chewy formulation, during an efficacy study) were analysed at four time points (4 h and 28, 56 and 84 days post-dosing) using an enantioselective analytical method. This method involved precipitation of 200 μl whole blood with acetonitrile and subsequent solid phase extraction (SPE) on C18 cartridges, evaporation to dryness and reconstitution in heptane/ethanol 4:6, *v*/*v*. Enantiospecific analysis was carried out by chiral normal phase HPLC using a Daicel Chiralpak IA-3 column (150 × 4.6 mm) and a mobile phase consisting primarily of heptane and isopropanol. Mass spectrometric detection was performed on an AB Sciex API 4000 Qtrap triple quadrupole instrument using the negative Turbo IonSpray ionisation mode and multiple reaction monitoring (MRM).

### Pharmacokinetic and statistical analysis

Pharmacokinetic parameters were calculated for individual animals using non-compartmental analysis. The validated statistical software SAS®, Version 9.2.2 was used for all calculations. The peak blood concentration (C_max_) and time to peak concentration (T_max_) were observed values, for the oral groups. The terminal half-life (T_1/2z_) was calculated by log-linear regression over a suitable time interval. The area under the concentration curve (AUC) between 0 and the last time point where the blood concentration was above the limit of quantitation (AUC_last_), was calculated by the linear trapezoidal rule and values below the limit of quantitation at the beginning of the profile were treated as zero. The area under the concentration curve from zero to infinity (AUC_inf_) was the sum of AUC_last_ and the extrapolation after the last observed timepoint; the second term was calculated by log-linear extrapolation from the last observed time point to infinity, using the half-life. The mean residence time (MRT) was calculated as the ratio of AUMC/AUC; where AUMC is the area under the first moment curve.

The clearance per kilogram of body weight (CL), defined as dose per kilogram of body weight/AUC, the volume of distribution at steady-state per kilogram of body weight (V_ss_), which is CL × MRT and the apparent volume of distribution per kilogram of body weight (V_z_), which is CL × T_1/2z_/ln(2), were determined for the intravenous group only.

Bioavailability (F%) in the oral groups was determined as (geometric mean of dose-normalized AUC_last_ in the oral group) / (geometric mean of dose-normalized AUC_last_ in the intravenous group). In this study, AUC_last_ was also equal to AUC from 0 to 35 days (AUC_0-35d_). AUC_inf_ was found to be an unsuitable parameter for the evaluation of bioavailability because it was not accurate due to the high percentage extrapolated beyond the last measured data point.

A one-way analysis of variance (ANOVA) was performed on log-transformed dose-normalized C_max_ and AUC parameters, with treatment as fixed effect. The mean and the 90% confidence interval (CI) for the difference between two treatment groups was calculated on the log scale and then back-transformed to the original scale, leading to the ratio between the two groups of C_max_ or AUC. The difference (on the log scale) between two treatment groups can be tested *versus* zero in a t-test (degrees of freedom given in subscripted parentheses after the symbol t in the tables; e.g. *t*_(21)_ meaning a *t*-value with 21 degrees of freedom).

### Translations

Spanish translation of the article is available in Additional file 1. French translation of the Abstract is available in Additional file 2.

## Results and discussion

### Enantiomeric stability in vivo

In 13 out of 16 adult dogs, no in vivo racemization was observed. In three dogs out of 16 animals, it was detectable only on day 84 but was negligible (less than 3%) and is believed to be of no clinical relevance for safety or efficacy. The absence of in vivo racemization in dogs after administration of pure enantiomer of lotilaner was clearly demonstrated. The absence of in vivo racemization is a prerequisite for a pure enantiomeric drug to make sense and for the investigation of the pharmacokinetics and safety of the opposite enantiomer to be omitted.

### Effect of feeding in dogs

**Fig. 2:**
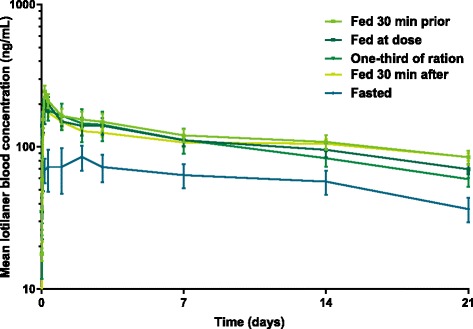
Dose-normalized (to 15 mg/kg) mean profile plots (± standard error) of lotilaner following oral administration under different feeding regimes

Since food may influence the pharmacokinetics and as feeding may facilitate treatment administration by the dog owner, the effect of feeding (time and amount) was evaluated in detail. Drug concentrations *versus* time profiles under the five tested feeding regimes are shown in Fig. 2. A pronounced feeding effect was found for lotilaner, however the exact time of feeding with respect to dosing (fed 30 min prior, fed at dosing, fed 30 min post-dosing) did not have a significant impact on bioavailability (see Table 1 for detailed test statistic and exact *P*-values). In addition, the reduction of the food ration to one-third of the daily ration also did not impact the bioavailability (Table 1). These findings offer a high degree of treatment flexibility to the dog owner, i.e. one-third of the daily ration is enough to provide adequate bioavailability and dosing can be performed at or around (± 30 min) the time of feeding. Hence, high bioavailability was found to be robust irrespective of changes in food amount and exact timing; moreover, it was achievable with both dry and wet food (unpublished data). However, fasted conditions (fed 5 h post-dose) yielded significantly lower bioavailability (Table 1), similarly to what was observed for fluralaner [19]. High bioavailability together with low between-animal variability is crucial in order to ensure reliable and robust efficacy, as lotilaner is a systemically acting ectoparasiticide and consequently blood concentrations are expected to be directly correlated with efficacy. Any individual case of low bioavailability would be expected to translate into the lower duration of efficacy.

**Table 1:** Effect of different feeding regimens on bioavailability: statistical comparisons (Study 1)

Treatment groups		Ratio^a^ (first/second)	90% CI	*t*-value	*P*-value^b^
First	Second				
Fed 30 min prior	Fed at dose	1.137	0.820–1.576	*t*_(20)_ = 0.68	0.5052
Fed 30 min prior	Fed 1/3 of ration	1.252	0.904–1.735	*t*_(20)_ = 1.19	0.2484
Fed 30 min prior	Fed 30 min after	1.029	0.743–1.426	*t*_(20)_ = 0.15	0.8799
Fed 30 min prior	Fasted	2.250	1.624–3.118	*t*_(20)_ = 4.29	**0.0004**
Fed at dose	Fed 1/3 of ration	1.101	0.795–1.526	*t*_(20)_ = 0.51	0.6154
Fed at dose	Fed 30 min after	0.905	0.653–1.255	*t*_(20)_ = -0.53	0.6050
Fed at dose	Fasted	1.979	1.428–2.742	*t*_(20)_ = 3.61	**0.0018**
Fed 1/3 of ration	Fed 30 min after	0.822	0.593–1.139	*t*_(20)_ = -1.04	0.3126
Fed 1/3 of ration	Fasted	1.797	1.297–2.490	*t*_(20)_ = 3.10	**0.0057**
Fed 30 min after	Fasted	2.186	1.577–3.029	*t*_(20)_ = 4.13	**0.0005**

^a^Ratio of bioavailabilities i.e. ratio of AUC_last_

^b^ *P*-values in bold denote significance

### Pharmacokinetic profile of lotilaner in dogs

**Fig. 3:**
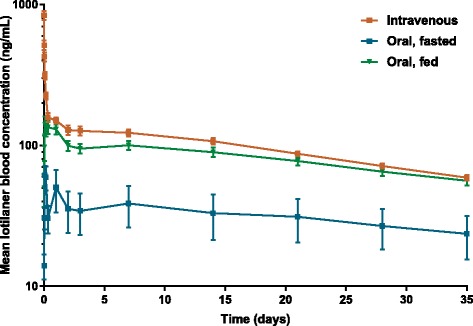
Dose-normalized (to 1 mg/kg) mean profile plots (± standard error) of lotilaner following intravenous or oral administration to fed or fasted dogs

The pharmacokinetic parameters of lotilaner are summarised in Table 2 and drug concentrations *versus* time profiles after intravenous and oral administration under fed or fasted conditions are shown in Fig. 3. The actual dose in the intravenous group ranged from 3.08 to 3.24 mg/kg, in the oral-fed group from 20.09 to 24.67 mg/kg, and in the oral-fasted group from 20.16 to 24.62 mg/kg. All pharmacokinetic parameters presented below are based on geometric means (considered as most appropriate, assuming that these parameters follow a log-normal distribution), except for T_max_ which can only take discrete values and is therefore based on the median.

**Table 2:** Mean ± standard deviation pharmacokinetic parameters of lotilaner in adult beagle dogs after either single administration at a target dose of 20 mg/kg orally to fasted dogs, orally to fed dogs, or at a target dose of 3.0 mg/kg intravenously

Parameter	Intravenous, 3 mg/kg (*n* = 8)	Oral, fed 20 mg/kg (*n* = 12)	Oral, fasted 20 mg/kg (*n* = 6)
Actual doses (mg/kg)	3.08–3.24	20.09–24.67	20.16–24.62
T_max_ (hours)	na	2 (range: 1–24)	4 (range: 2–24)
C_max_ (ng/ml)	na	4011 ± 990	1454 ± 849
AUC_last_ (day*ng/ml)	10,976 ± 1381	62,840 ± 17,346	18,592 ± 16,632
AUC_inf_ (day*ng/ml)	17,844 ± 1937	118,600 ± 42,287	40,886 ± 31,915
C_max_ (dose normalized^a^) (ng/ml)	na	179.2 ± 43.5	65.4 ± 35.7
AUC_last_ (dose normalized^a^) (day*ng/ml)	3436 ± 430	2806 ± 801	837 ± 731
AUC_inf_ (dose normalized^a^) (day*ng/ml)	5586 ± 666	5297 ± 1921	1840 ± 1406
T_1/2z_ (day)	24.6 ± 5.9	30.7 ± 10.0	38.8 ± 11.2
MRT (day)	36.0 ± 8.5	45.3 ± 13.9	56.9 ± 14.8
CL (l/kg/day)	0.18 ± 0.02	na	na
V_z_ (l/kg)	6.35 ± 1.27	na	na
V_ss_ (l/kg)	6.45 ± 1.26	na	na
Bioavailability (F%)	na	81.7	24.3

^a^Dose-normalized to 1 mg/kg

All values (Mean and standard deviation) are based on geometric summary statistics (corresponding to summary statistics of log transformed values and then back-transformed), except for T_max_ which is based on the median

After intravenous administration at 3 mg/kg, a visual inspection of the profiles showed that lotilaner blood concentrations decreased bi-exponentially with a rapid distribution phase and a long elimination phase. The terminal half-life of lotilaner was 24.6 days and MRT was 36.02 days. Total blood clearance was 0.18 l/kg/day and the volumes of distribution V_z_ and V_ss_ were 6.35 and 6.45 l/kg, respectively. Mean dose-normalized AUC_last_ was 3436 day*ng/ml.

After oral administration at 20 mg/kg, a visual inspection of the profiles showed that lotilaner blood concentrations decreased bi-exponentially after T_max_, with a rapid distribution phase within the first day of administration and a long elimination phase. After oral administration in fed conditions, detectable blood levels were identified in most treated dogs within 30 min and concentrations peaked quickly (mean dose-normalized C_max_ of 179 ng/ml) with a T_max_ at 2 h, indicating rapid dissolution and absorption of the chewable tablet. The terminal half-life was 30.7 days and MRT was 45.3 days. Mean dose-normalized AUC_last_ (= AUC_0-35d_) was 2806 day*ng/ml. After oral administration in fasted conditions, lower blood concentrations of lotilaner were observed with a mean dose-normalized C_max_ of 65 ng/ml. T_max_ was observed later, at 4 h. The terminal half-life was 38.7 days and MRT was 56.9 days. Mean dose-normalized AUC_last_ (= AUC_0-35d_) was 837 day*ng/ml. The mean terminal half-life after oral administration was in the same range as the one determined after intravenous administration, indicating that the terminal phase represented the true elimination phase.

For comparisons from the oral-fed and oral-fasted groups, differences in mean values for C_max_, AUC_last_, AUC_inf_ were significant and the difference in bioavailability (Table 2) between the oral-fed (81.7%) and oral-fasted groups (24.3%) was also significant (see Table 3 for detailed test statistic and exact *P*-values). The differences in half-life and mean residence time between fed and fasted states were not significant (Table 3). Moreover, the variability of the lotilaner pharmacokinetic parameters was moderate in the intravenous and oral-fed groups and much higher in the oral-fasted group. This high variability in the oral-fasted group was explained by the low bioavailability since this is known as a major source of variability [20].

**Table 3:** Comparisons between fed and fasted groups following oral administration of lotilaner at 20 mg/kg (Study 2)

Parameter	Ratio (fasted to fed)	90% CI	*t*-value	*P*-value^a^
T_1/2z_	1.263	0.983–1.624	*t*_(23)_ = -1.59	0.1244
MRT	1.256	0.991–1.591	*t*_(23)_ = -1.65	0.1128
C_max_ (dose normalized)	0.365	0.265–0.502	*t*_(16)_ = 5.51	**< 0.0001**
AUC_last_ (dose normalized)	0.298	0.201–0.441	*t*_(23)_ = 5.29	**< 0.0001**
AUC_inf_ (dose normalized)	0.347	0.238–0.507	*t*_(23)_ = 4.80	**< 0.0001**
Bioavailability	0.298	0.201–0.441	*t*_(23)_ = 5.29	**< 0.0001**

^a^ *P*-values in bold denote significance

In order to interpret the clearance, the overall body extraction ratio (which can be regarded as the percentage of drug being cleared by the entire body during a single passage through the clearing organs) was computed by the body clearance (0.18 l/kg/day) divided by the cardiac output (approximately 167 l/kg/day for a 10 kg dog) [21]. Hence, the total blood clearance corresponded to an overall extraction ratio of 0.1% and is therefore considered as very low. In addition, lotilaner had high volumes of distribution (> 6 l/kg), as expected for a lipophilic drug that would distribute in the fatty tissue. The low clearance combined with the large volume of distribution explains the long half-life of lotilaner in the dog [22, 23]. As compared to the other isoxazolines developed for dogs (afoxolaner, fluralaner and sarolaner), lotilaner in the present study had the longest half-life (approximately 4 weeks *versus* 2 weeks for the other compounds). This difference was mainly explained by the largest volume of distribution (approximately 6 l/kg for lotilaner *versus* 3 l/kg for the other compounds), whereas the clearance was in the same range (from 0.12 to 0.18 l/kg/day) [6, 8, 24]. This long terminal half-life and mean residence time explains the persistent systemic availability of lotilaner and provides effective blood concentrations for the entire duration of the inter-dosing interval of 1 month. Variability in half-life between individuals or between studies and populations was observed in the numerous studies performed during the development program, however, care was taken during dose characterization to select a robust dose high enough to provide 1 month duration of efficacy even in individuals with a shorter half-life and at the lowest possible therapeutic dose within the dose band.

The feeding effect on pharmacokinetics was multiple, not only did administration in fasted dogs lead to much lower bioavailability, but also to a delayed T_max_ and to an increase between-animal variability. Consequently, administration of lotilaner to fasted dogs is not recommended. Achievement of the maximum blood concentration within 2 h following lotilaner administration to dogs in the fed state aligns with the demonstrated rapid onset of adulticidal (flea and tick) activity [12–16]. Similarly, the demonstrated long half-life of lotilaner (30.7 days in the oral-fed group) and the sustained concentration levels above the estimated flea- and tick-lethal breakpoints through at least 1 month align with the prolonged effectiveness observed in multiple studies in which challenge with these parasites was extended through 35 days after lotilaner treatment.

## Conclusions

The pharmacokinetic properties of lotilaner (Credelio™) were investigated in a number of studies. Following a single intravenous administration, lotilaner had a very low clearance (0.18 l/kg/day) and high volumes of distribution (> 6 l/kg), resulting in a long terminal half-life (24.6 days). Following a single oral administration to fed dogs, lotilaner blood concentrations peaked within 2 hours, had a long half-life of 30.7 days, and had significantly greater bioavailability than when administered to fasted dogs. Food (at least 1/3 of the daily ration) enhanced the bioavailability to > 80% and therefore it is recommended to administer chewable flavoured tablets at or around the time of feeding. Hence, lotilaner administered to fed dogs at a minimum dose of 20 mg/kg should, therefore, provide a rapid onset of flea and tick killing activity, with consistent and sustained effectiveness for at least 1 month after treatment.

## Additional files

Additional file 1: Spanish translation of the article. (PDF 148 kb)

Additional file 2: French translation of the Abstract. (PDF 17 kb)

## Abbreviations

ANOVA: Analysis of variance
AUC_inf_: Area under the blood concentration-time curve from zero to infinity
AUC_last_: Area under the blood concentration-time curve from zero to the last time point for which blood concentration is above the limit of quantitation
CI: Confidence interval
CL: Total body clearance of drug from the blood
C_max_: Maximum (peak) blood drug concentration
EMA: European Medicine Agency
FDA: Food and Drug Administration
GABACls: γ-aminobutyric acid (GABA)-gated chloride channels
LC-MS/MS: Liquid chromatography tandem mass spectrometry
LLOQ: Lower limit of quantification
na: Not applicable
T_1/2z_: Elimination half-life associated with the terminal slope of a semi-logarithmic concentration time curve
T_max_: Time to reach maximum (peak) blood concentration following drug administration
V_ss_: Volume of distribution at steady-state
V_z_: Volume of distribution during the terminal phase
