# Supplementary material for: The intravenous and oral pharmacokinetics of lotilaner in dogs
Source: Parasit Vectors. 2017 Nov 1;10:522. doi: 10.1186/s13071-017-2475-z (PMC5664907; doi:10.1186/s13071-017-2475-z)
Supplement: Supplementary file 1 — Spanish translation of the article. (PDF 148 kb) [file 13071_2017_2475_MOESM1_ESM.pdf]

La farmacocinética intravenosa y oral de lotilaner en perros

Céline E. Toutain[1]*, Wolfgang Seewald[1] y Martin Jung[1]

[1]Elanco Animal Health, Mattenstrasse 24a, CH-4058, Basel, Switzerland

*Correspondencia: celine.toutain@elanco.com

E-mails:

CET: celine.toutain@elanco.com

WS: wolfgang.seewald@elanco.com

MJ: martin.jung@elanco.com

Resumen

Antecedentes: Lotilaner es un nuevo ectoparasiticida oral de la clase de la isoxazolina desarrollada para el tratamiento contra las infestaciones de pulgas y garrapatas en perros. Está formulada como un *S*-enantiómero puro en tabletas masticables saborizadas (Credelio™). La farmacocinética de lotilaner fue determinada completamente después de la administración intravenosa y oral y bajo diferentes regímenes de alimentación en perros.

Métodos: Se enrolaron veintiséis perros Beagle adultos en un estudio de farmacocinética evaluando ya sea la administración intravenosa u oral de lotilaner. Se colectaron muestras de sangre hasta 35 días después de la administración oral de 20 mg/kg, bajo condiciones de subalimentación o ayuno, o administración intravenosa de 3 mg/kg. Los efectos de los tiempos en que se ofreció alimento y la cantidad de alimento consumido antes o después de la dosis sobre la biodisponibilidad se evaluaron en un estudio por separado en 25 perros adultos. Las concentraciones de sangre de lotilaner se midieron usando el método de cromatografía líquida validada/espectrometría de masas en tándem (LC-MS/MS). Los parámetros farmococinéticos se calcularon por análisis no compartamental.. Además se evaluó *in vivo* la estabilidad del enantiómero en un estudio analítico.

Resultados: Después de la administración oral en animales alimentados, lotilaner fue absorbido inmediatamente y alcanzó concentraciones pico en sangre al cabo de 2 h. La vida media terminal fue de 30.7 días. El alimento mejora la absorción, proporcionando una biodisponibilidad oral por arriba

del 80% y redujo la variabilidad inter-individual. Más aún, el tiempo de alimentación con respecto a la dosificación (alimentados 30 minutos antes, alimentados al momento de la dosificación o alimentados a los 30 minutos post-dosificación) o la reducción de la ración de alimento a un tercio de la ración diaria normal no impactó la biodisponibilidad. Después de la administración intravenosa, lotilaner tuvo una eliminación baja de 0.18 l/kg/día, grandes volúmenes de distribución V_z y V_{ss} de 6.35 y 6.45 l/kg, respectivamente, y una vida media terminal de 24.6 días. Además, no hubo una racemización *in vivo* lotilaner.

Conclusiones: Se estudiaron en detalle las propiedades farmacocinéticas de lotilaner administrado oralmente como una tableta masticable saborizada (Credelio^{TM}). Con un T_{max} de 2 h y una vida media terminal de 30.7 días bajo condiciones de alimentación, lotilaner provee un rápido inicio de la actividad de matar a las pulgas y garrapatas con una eficacia consistente y sostenida por al menos un mes.

Palabras clave: Lotilaner, Isoxazolina, Farmacocinética, Perro, Oral, Intravenosa, Efecto del alimento, Alimentados, Ayunados

Antecedentes

La infestación con pulgas y garrapatas es un desafío de salud indiscutible para los perros en todo el mundo, ya que pueden causar una gran variedad de condiciones dermatológicas y anemia. Las pulgas hembra comienzan a poner huevos al cabo de 24-36 h después de adquirir a un hospedador, pueden producir tantos como 40 a 50 huevos por día [1], creando rápidamente una infestación. Mientras que la mayoría de las garrapatas de 3-hospedadores que infestan a los perros son adquiridas del ambiente externo y requieren de hospedadores adicionales, *Rhipicephalus sanguineus* (*sensu lato*) (*s.l.*) prefieren alimentarse de perros durante las etapas larva, de ninfa y adulta y van a infestar casas, perreras y hospitales veterinarios. El ciclo de vida de *R. sanguineus* (*s.l.*) puede ser completado completamente en interiores en tan poco tiempo como 2 meses bajo condiciones ideales [2]. Adicionalmente, las pulgas y las garrapatas son vectores competentes para varias enfermedades infecciosas e infecciones endoparasitarias secundarias en perros y en agentes de enfermedad transmitidos por vectores que pueden ser transmitidos a las personas o a otras especies animales [3]. Existen reportes anecdóticos que las poblaciones ectoparasitarias con una sensibilidad reducida a productos viejos para pulgas y garrapatas están aumentando [4]. Basándose en estos problemas, es

importante encontrar nuevas formas de manejar rápidamente tanto las infestaciones de pulgas, como de garrapatas.

Las isoxazolinas son una nueva familia de compuestos que ha demostrado tener actividad contra pulgas y garrapatas [5-7]. Se monitorearon unos compuestos de una biblioteca de más de 1000 estructuras contra insectos y ácaros *in vitro* y en estudios con roedores. El candidato líder, lotilaner, i.e. (*S*)-5-[5-(3,4,5-tricloro-fenil)-5-trifluorometil-4,5-dihidroisoxazol-3-il]-3-metil-tiofeno-2-ácido carboxílico [(2,2,2-trifluoro-etilcarbamoil)-metil]-amida (Fig. 1) surgió como el candidato más apropiado cuando se monitoreo para tanto eficacia, como seguridad. Tiene un peso molecular de 596.76, un logaritmo Pow(coeficiente de repartición octanol/agua) medido de 5.3. Lotilaner, como sarolaner [6] o afoxolaner [8], se une fuertemente a las proteínas plasmáticas en el perro (datos no publicados). Con un centro quiral, con enantiómeros *R*- y *S*- que pueden existir, y siendo que el *S*- enantiómero es lotilaner. Se midió *in vitro* una diferencia en potencia de 10-100 veces entre lotilaner y su enantiómero opuesto (*R*-enantiómero) contra *Ctenocephalides felis* y *R. sanguineus* (*s.l.*) (datos no publicados), Credelio^{TM} fue desarrollado como un *S*-enantiómero puro. Esto reduce la cantidad de ingrediente activo en la formulación final, elimina la necesidad de un compuesto inactive para ser metabolizado y/o excretado por el paciente y limita la posibilidad de los efectos secundarios farmacodinámicos. En otras palabras, la misma dosis, si se administra como *S*-enantiómero puro, proporciona una mejor eficacia y un período de protección más prolongado en comparación con el racemato, sabiendo que no hay racemización *in vivo*.

Las isoxazolinas son inhibidores potentes de ácido γ-aminobutirico (GABA)-compuertas de los canales de cloro (GABACls) [9, 10]. El influjo del GABA-mediado por cloro lleva a la hiperpolarización de la membrana celular y genera un potencial post-sináptico inhibitorio, que disminuye la probabilidad de una acción potencial. Los insectos y otros invertebrados poseen GABACls que no solamente son expresados en el sistema nervioso central, en donde generan potenciales inhibitorios para la integración correcta de las señales neuronales, sino también en sitios neuromusculares periféricos, en donde promueven la relajación muscular. En la presencia de lotilaner, los GABACls no son capaces de abrirse ante la estimulación del GABA, definiendo esta molécula como un antagonista de los GABACls. Los parásitos expuestos a lotilaner experimentan una parálisis espástica llevándolos a su inanición y muerte (datos no publicados). Además, se ha probado que las isoxazolinas son específicas para los neurorreceptores de los insectos y ácaros, más que para los neurorreceptores de los mamíferos. La falta de efecto sobre el sistema nervioso de un mamífero en dosis clínicamente relevantes fue confirmado en varios estudios de seguridad en laboratorio y en animales blanco. Dosis elevadas repetidas administradas oralmente en intervalos de

cuatro semanas a perros jóvenes a las 8 semanas de edad demostraron que el nivel de dosis mínima de 43 mg/kg/mes de lotilaner tiene un amplio margen de seguridad [11].

A una dosis de 20 mg/kg, se ha demostrado que lotilaner provee un inicio rápido de la actividad contra las pulgas y garrapatas que es sostenida, al menos, por un mes después del tratamiento [12-16]. Lotilaner está formulado como tabletas masticables saborizadas (Credelio™) y se debe administrar por vía oral mensualmente. Para poder entender las propiedades de lotilaner, se llevaron a cabo estudios de determinación de la dosis de apoyo y desarrollo de la formulación en perros adultos para determinar el perfil farmacocinético después de la administración intravenosa y oral, y para describir el efecto de la alimentación sobre los parámetros farmacocinéticos.

Métodos

Manejo del animal

Se utilizaron perros Beagle adultos, de ambos sexos criados para este propósito y que pesaban entre 9 y 16 kg. Cada animal fue identificado de manera única y se aclimatizaron a las condiciones del estudio por al menos 1 semana. Sólo los animales sanos fueron incluidos y se evaluó que fueran adecuados por medio de un examen físico y patología clínica. Los perros se alojaron en instalaciones interior con clima controlado de acuerdo con lo aceptado para el cuidado de animales de laboratorio y las guías de uso. Se mantuvieron en pequeños grupos excepto por los días alrededor de la administración del tratamiento, en donde los perros se alojaron individualmente por al menos 1 día, para evitar una potencial contaminación cruzada entre animales. A los perros se les dio la oportunidad de hacer ejercicio en el exterior y de tener interacción social diariamente. Se alimentaron diariamente con una ración apropiada de un alimento comercial canino y agua disponible *ad libitum*. Se observaron los perros para determinar su salud general, comportamiento y apetito al menos una vez al día al o largo de los estudios. Todos los animales retornaron a sus instalaciones de alojamiento normales al completar los estudios.

Diseños experimentales

Estudio 1

En el primer estudio, diseñado para investigar el efecto de la alimentación sobre la farmacocinética de lotilaner después de la administración oral, 25 perros adultos de ambos sexos se ubicaron en cinco grupos de tratamiento con cinco perros en cada grupo. Cada perro recibió una sola

administración oral de una tableta cercana a la formulación final, a una dosis blanco de 15 mg/kg de lotilaner (la dosis terapéutica que se pretendía tener inicialmente). Los perros se ayunaron la noche anterior y se probaron cinco regímenes de alimentación diferentes como sigue: los perros recibieron su ración completa de alimento diaria (i) 30 minutos antes a, (ii) al mismo tiempo, (iii) 30 minutos después, (iv) 5 h después de la administración del tratamiento o (v) solamente un tercio de su ración completa de alimento diaria al mismo tiempo que la administración del tratamiento. Se colectaron muestras de sangre de la vena yugular en tubos de K3-EDTA pre-dosis y a 30 minutos, a 1, 2, 4, 8, 24, 48 y 72 h y 7, 14 y 21 días post-tratamiento.

Estudio 2

En el segundo estudio, en donde se pretendía determinar el perfil farmacocinético de lotilaner después de la administración intravenosa y oral, se ubicaron 26 perros adultos de ambos sexos en tres grupos de tratamiento como sigue: un grupo de ocho perros intravenoso, un grupo oral de 12 perros tratados 30 (±5) minutos después de la alimentación, y un grupo de seis perros en ayuno. Cada perro de los grupos orales recibió una sola administración de la formulación final de la tableta (conteniendo 35% p/p de lotilaner), a la dosis blanco mínima de 20 mg/kg de lotilaner, que fue la dosis terapéutica final. Cada perro del grupo intravenoso recibió una sola administración de lotilaner en una solución que consistía de 23% p/v de solución salina fisiológica y de tetraglicol agregado 100% p/v, a la dosis blanco de 3 mg/kg de lotilaner, que es una dosis conocida que es bien tolerada intravenosamente. Las muestras de sangre colectadas de la vena yugular en tubos K3-EDTA a la pre-dosis y a 5 minutos (intravenoso solamente), 30 minutos, a 1, 2, 4, 8, 24, 48 y 72 h y 7, 14, 21, 28 y 35 días post-tratamiento.

Para ambos estudios, las muestras de sangre complete se almacenaron congeladas (por un máximo de 5 meses) a aproximadamente -20°C hasta su análisis con un método validado LC-MS/MS. Durante la validación, la estabilidad en las condiciones de almacenamiento se demostraron por al menos 9 meses.

Análisis de lotilaner en sangre

Se analizó cuantitativamente lotilaner en sangre usando un método analítico involucrando la cromatografía líquida con detección en espectrometría de masas en tándem (LC-MS/MS). Se extrajeron muestras de sangre completa de perro (80 µl) por precipitación con acetonitrilo y se diluyeron más aún con actonitrilo. Se utilizó un análogo químico relacionado cercanamente como un estándar interno. En cada 10 µl de sobrenadante diluido se realizó cromatografía por HPLC en

columna de fase inversa [Thermo Betasil C18, 5 µm (50 × 4.6 mm)] con una fase móvil isocrática que consistía de 0.1 % de ácido fórmico y acetonitrilo (15:85, v:v) usando una tasa de flujo de 0.8 ml/min y se analizó cuantitativamente en un Sistema de espectrómetro de masas AB Sciex API 5000 o API 5500 triple cuadripolar usando el modo de ionización negativo Turbo IonSpray y el monitoreo de reacción múltiple (MRM) de la transición m/z 596 → 181 para lotilaner.

El método se validó sobre un rango lineal de 6.8 a 6800 ng/ml, con un límite menor de cuantificación (LLOQ) de 6.8 ng/ml, de acuerdo a las guías del FDA y EMA [17, 18]. La precisión media inter-día fue de 14.9% a LLOQ y tuvo un rango entre 3.4 y 7.8 % en otros niveles y la precisión media inter-día tuvo un rango entre 100.3 y 103.6 %. Además, se establecieron la especificidad, la integridad de la dilución, la recuperación y el efecto matriz, residuos y estabilidad en la matriz y las soluciones. La estabilidad a largo plazo en sangre congelada a -20 °C fue demostrada a lo largo de 9 meses.

Estabilidad enantiomérica

La estabilidad enantiomérica *in vivo* de lotilaner se investigó en un estudio analítico. Las muestras de sangre de 16 perros adultos que recibieron una sola dosis oral del enantiómero puro del fármaco a razón de 15 mg/kg (tableta o formulación masticable, durante un estudio de eficacia) se analizaron en cuatro puntos en el tiempo (4 h y 28, 56 y 84 días post-dosificación) usando un método analítico enantioselectivo. Este método involucró la precipitación de 200µl de sangre completa con acetonitrilo y la subsecuente extracción de la fase sólida (SPE) en cartuchos C18, evaporación a desecación y reconstitución en heptano/etanol 4:6, v/v. El análisis enantioespecífico se llevó a cabo por la fase quiral normal en HPLC usando una columna Daicel Chiralpak IA-3 (150 × 4.6 mm) y una fase móvil que consistía principalmente de heptano e isopropanol. La detección espectrométrica de masas se llevó a cabo en un instrumento AB Sciex API 4000 Atrapa triple cuadripolar usando el modo de la ionización negativa Turbo IonSpray y la reacción de monitoreo múltiple (MRM).

Análisis farmacocinético y estadístico

Los parámetros farmacocinéticos se calcularon para los animales individualmente usando un análisis no compartamental. El programa estadístico validado SAS®, Versión 9.2.2 fue el utilizado para todos los cálculos. La concentración pico en sangre (C_{max}) y el tiempo de la concentración pico (T_{max}) fueron valores observados para todos los grupos orales. La vida media terminal ($T_{1/2z}$) se calculó por medio de una regresión lineal-logarítmica sobre el intervalo adecuado. El área bajo la

curva de concentración (AUC) entre 0 y el último punto en donde la concentración en sangre estaba arriba del límite de cuantificación ($Aus_{cúltame}$) fue calculado por la regla trapezoidal lineal y los valores bajo el límite de cuantificación al inicio del perfil fueron tratados como cero. El área bajo la curva de concentración de cero a infinito (AUC_{inf}) fue la suma de la $AUC_{última}$ y la extrapolación después del último punto en el tiempo observado; el Segundo plazo se calculó por medio de extrapolación logarítmica-lineal a partir del ultimo punto en el tiempo observado al infinito, usando la vida media. El tiempo medio de residencia (MRT) se calculó como la proporción de AUMC/AUC; en donde AUMC es el área bajo el primer momento de la curva.

La eliminación por kilogramo de peso corporal (CL), definida como dosis por kilogramo de peso corporal/AUC, el volumen de la distribución en estado-estático por kilogramo de peso corporal (V_{ss}), que es CL x MRT y el volumen aparente de distribución por kilogramo de peso corporal (V_z), que es CL x $T_{1/2z}$ ⁄ en(2), fueron determinados para el grupo intravenoso solamente.

La biodisponibilidad (F%) en los grupos orales fue determinada como (la media geométrica de la dosis-normalizada $AUC_{última}$ en el grupo oral) / (la media geométrica de la dosis-normalizada $AUC_{última}$ en el grupo intravenoso). En este estudio, el $AUC_{último}$ fue también igual al AUC de 0 a 35 días ($AUC_{0\text{-}35d}$). Se encontró que el AUC_{inf} no es un parámetro adecuado para la evaluación de la biodisponibilidad debido a que no es preciso debido al alto porcentaje extrapolado más allá del último punto de dato medido.

Se llevó a cabo un análisis de varianza de una-vía (ANOVA) en una dosis-normalizada logarítmicamente transformada a C_{max} y parámetros AUC, con el tratamiento como un efecto fijo. La media y el intervalo de confianza del 90% (CI) para la diferencia entre los dos grupos de tratamiento se calculó en la escala-logarítmica y luego se retro-transformó en la escala original, llevando a una proporción entre los dos grupos de C_{max} o AUC. La diferencia (en la escala logarítmica) entre dos grupos de tratamiento puede ser probada versus cero en una prueba-t (grados de libertad dados en paréntesis subscritos después del símbolo t en los cuadros; e.g. $t_{(21)}$ que significa un valor-t con 21 grados de libertad).

Resultados y discusión

Estabilidad enantiomérica *in vivo*

En 13 de 16 perros adultos no se observe la racemización *in vivo*. En tres perros de 16 animales, solamente se detectó en el día 84 pero fue nula (menos del 3%) y se cree que no tiene relevancia

clínica para la seguridad o la eficacia. Se demostró claramente la ausencia de la racemización *in vivo* en perros después de la administración del enantiómero puro de lotilaner. La ausencia de la racemización *in vivo* es un prerrequisito para que el fármaco enantiomérico puro haga sentido y para que la investigación de la farmacocinética y la seguridad del enantiómero opuesto sea omitida.

Efecto de la alimentación en perros

Puesto que el alimento puede influir en la farmacocinética y como la alimentación puede facilitar la administración del tratamiento para el propietario del perro, se evaluó en detalle el efecto de la alimentación (tiempo y cantidad). En la Fig. 2 se muestran las concentraciones del fármaco vs. los perfiles de tiempo, bajo los cinco regímenes de alimentación probados. Se encontró un pronunciado efecto de la alimentación para lotilaner, sin embargo el tiempo exacto de la alimentación con respecto a la dosis (alimentado 30 minutos antes, alimentado al momento de la dosificación, alimentado 30 minutos post-dosificación) no tuvo un impacto significativo sobre la biodisponibilidad (ver Tabla 1 para la prueba estadística detallada y los valores-*P* exactos). Además, la reducción de la ración de alimento tercio de la ración diaria tampoco impactó la biodisponibilidad (Tabla 1). Estos hallazgos ofrecieron un alto grado de flexibilidad en el tratamiento para el propietario del perro, i.e. un tercio de la ración diaria es suficiente para proveer una biodisponibilidad adecuada y la dosificación puede llevarse a cabo a o alrededor (± 30 min) el momento de la alimentación. Por ende, la elevada biodisponibilidad se encontró como robusta independientemente de los cambios en la cantidad de alimento y los tiempos exactos; más aún, fue alcanzable con alimento seco y húmedo (datos no publicados). Sin embargo, las condiciones de ayuno (alimentados 5 h post-dosificación) produjo una biodisponibilidad significativamente más baja (Tabla 1), de manera similar a lo que se observe con fluralaner [19]. La elevada biodisponibilidad junto con la baja variabilidad-entre-animales son cruciales para poder asegurar la eficacia confiable y robusta, como lotilaner es un ectoparasiticida de acción sistémica y consecuentemente las concentraciones en sangre se espera que estén directamente correlacionadas con la eficacia. Cualquier caso individual de baja biodisponibildad se esperaría que se tradujera en una duración más baja de la eficacia.

Perfil farmacocinético de lotilaner en perros

Los parámetros farmacocinéticos de lotilaner se resumen en el Tabla 2 y las concentraciones del fármaco vs. los perfiles de tiempo después de la administración intravenosa y oral bajo condiciones de alimentación o ayuno se muestran en la Figura 3. La dosis real en el grupo intravenoso tuvo un

rango de 3.08 a 3.24 mg/kg, en el grupo alimentado-oral de 20.09 a 24.67 mg/kg, y en el grupo ayunado-oral de 20.16 a 24.62 mg/kg. Todos los parámetros farmacocinéticos presentados a continuación se basan en las medias geométricas (consideradas como las más apropiadas, asumiendo que estos parámetros siguieron una distribución logarítmica-normal), excepto para T_{max} que solamente puede tomar valores discretos y por lo tanto se basa en la mediana.

Después de la administración intravenosa a 3 mg/kg, una inspección visual de los perfiles mostraron que las concentraciones en sangre de lotilaner disminuyeron vi-exponencialmente con una fase de distribución rápida y una fase de larga eliminación. La vida media terminal de lotilaner fue de 24.6 días y la MRT fue de 36.02 días. La eliminación total en sangre fue de 0.18 l/kg/día y los volúmenes de distribución V_z y V_{ss} fueron de 6.35 y 6.45 l/kg, respectivamente. La dosis media-normalizada $AUC_{última}$ fue de 3436 día*ng/ml.

Después de la administración oral a 20 mg/kg, una inspección visual de perfiles mostró que las concentraciones en sangre de lotilaner disminuyeron bi-exponencialmente después del T_{max}, con una fase de distribución rápida dentro del primer día de administración y una fase larga de eliminación. Después de la administración oral en condiciones de alimentación, los niveles en sangre detectables fueron identificados en la mayoría de los perros tratados al cabo de 30 min y las concentraciones llegaron al pico rápidamente (dosis media-normalizada C_{max} de 179 ng/ml) con un T_{max} de 2 h, indicando la disolución y absorción rápidas de la tableta masticable. La vida media terminal fue de 30.7 días y un MRT de 45.3 días. La dosis media-normalizada $AUC_{última}$(=$AUC_{0\text{-}35d}$) fue de 2806 día*ng/ml. Después de la administración oral en condiciones de ayuno, las concentraciones de lotilaner se observaron con una dosis media-normalizada C_{max} de 65 ng/ml. El T_{max} se observó más tarde, a las 4 h. La vida media terminal fue de 38.7 días y la MRT fue de 56.9 días. La dosis media-normalizada $AUC_{última}$(=$AUC_{0\text{-}35d}$) fue de 837 día*ng/ml. El promedio de la vida media terminal después de la administración oral estuvo en el mismo rango como el determinado para la administración intravenosa, indicando que la fase terminal representa la verdadera fase de eliminación.

Para las comparaciones de los grupos alimentado-oral y ayunado-oral, las diferencias en los valores medios para C_{max}, $AUC_{última}$, AUC_{inf} fueron significativas y la diferencia en biodisponibilidad (Tabla 2) entre los grupos alimentado-oral (81.7%) y ayunado-oral (24.3%) también fue significativo (ver el Tabla 3 para la prueba estadística detallada y los valores-*P* exactos). Las diferencias en la vida media y el tiempo de residencia medio entre los estados de alimentación y ayuno no fueron significativas (Tabla 3). Más aún, la variabilidad de los parámetros farmacocinéticos de lotilaner fue moderada en los grupos intravenoso y alimentado-oral y mucho

más en el grupo ayunado-oral. Esta alta variabilidad en el grupo ayunado-oral se explicó por la baja biodisponibilidad puesto que esto se conoce como una fuente principal de variabilidad [20].

Para poder interpretar la eliminación, la proporción de la extracción general del cuerpo (que puede ser considerada como el porcentaje del fármaco que se está eliminando de todo el cuerpo durante un solo paso a través de los órganos de eliminación) fue computada por la eliminación corporal (0.18 l/kg/día) dividida por el impulso cardíaco (aproximadamente 167 l/kg/día para un perro de 10 kg) [21]. Por ende, la eliminación total de la sangre corresponde a la proporción de extracción general de 0.1% y por lo tanto se considera como muy baja. Además, lotilaner tuvo altos volúmenes de distribución (> 6 L/kg), como se espera para el fármaco liofilice que se distribuye en el tejido adiposo. La baja eliminación combinada con el gran volumen de distribución explica la larga vida media de lotilaner en el perro [22, 23]. En comparación con otras isoxazolinas desarrolladas para perros (afoxolaner, fluralaner y sarolaner), en el presente estudio, lotilaner tuvo la vida media más larga (aproximadamente, 4 semanas vs 2 semanas para los otros compuestos). Esta diferencia se explicó principalmente por el volumen de distribución más grande (aproximadamente 6 l/kg para lotilaner vs 3 l/kg para los otros compuestos), mientras que la eliminación estuvo en el mismo rango (de 0.12 a 0.18 l/kg/día) [6, 8, 24]. Esta vida medio terminal más larga y el tiempo de residencia medio explican la disponibilidad sistémica persistente de lotilaner y proveen concentraciones efectivas en sangre para toda la duración del intervalo inter-dosificación de un mes. La variabilidad de la vida media entre individuos o entre estudios y poblaciones se observa en varios estudios llevados a cabo durante el programa de desarrollo, sin embargo, se tuvo cuidado durante la caracterización de la dosis para seleccionar una dosis elevada robusta suficiente para proveer una duración de eficacia de un mes aún en individuos con una vida media más corta y a la dosis terapéutica más baja posible dentro de la banda de la dosis.

El efecto de alimentación sobre la farmacocinética fue múltiple, no solo la administración en perros en ayuno llevó a una biodisponibilidad mucho menor, sino también a un T_{max} y a un incremento de variabilidad entre animales. Consecuentemente, no se recomienda la administración de lotilaner para perros en ayuno.

El logro de la concentración máxima en sangre al cabo de 2 h después de su administración a perros alimentados se alinea con el inicio rápido de la actividad adulticida (pulgas y garrapatas) [12-16]. De manera similar, la vida media larga de lotilaner (30.7 días en el grupo alimentado-oral) y los niveles de concentración sostenidos arriba de los puntos de quiebre letales para pulga- y garrapata- a través de al menos 1 mes se alinean con la efectividad prolongada observada en

estudios múltiples en donde el desafío con estos parásitos se extendió a través de 35 días después del tratamiento con lotilaner.

Conclusiones

Las propiedades farmacocinéticas de lotilaner (Credelio^{TM}) se investigaron en varios estudios. Después de una sola administración intravenosa, lotilaner tuvo una eliminación muy baja (0.18 l/kg/día) y altos volúmenes de distribución (> 6 l/kg), resultando en una vida media terminal larga (24.6 días). Después de una sola administración oral a perros alimentados, las concentraciones en sangre de lotilaner llegaron al pico al cabo de 2 h, tuvieron una vida media larga de 30.7 días, y una biodisponibilidad significativamente mayor que cuando se administró a perros en ayuno. El alimento (al menos 1/3 de la ración diaria) mejoró la biodisponibilidad a >80% y por lo tanto se recomienda administrar las tabletas masticables saborizadas a o alrededor del momento de la alimentación. De ahí que, lotilaner administrado a perros alimentados a una dosis mínima de 20 mg/kg deberá de proveer un inicio rápido de la actividad de matar a pulgas y garrapatas, con una efectividad consistente y sostenida por al menos un mes después del tratamiento.

Abreviaturas

ANOVA: Análisis de Varianza;AUCinf: Área bajo la curva de tiempo-concentración de sangre de cero a infinito; AUCúltima. Área bajo la curva de tiempo-concentración de cero al último punto de tiempo en donde la concentración de sangre está arriba del límite de cuantificación; CI: Intervalo de Confianza; Cmax: Concentración máxima (pico) de un fármaco en sangre; CL: Eliminación total del cuerpo de un fármaco en sangre; EMA: Agencia Europea de Medicina; FDA: Administración de Alimentos y Fármacos; GABACls: ácido γ-aminobutírico (GABA)-compuertas de canales de cloro; LC-MS/MS: Cromatografía Líquida en tándem con Espectrometría de Masas; LLOQ: Límite más bajo de cuantificación; Tmax: Tiempo para alcanzar la concentración máxima (pico) en sangre después de la administración de un fármaco; T1/2z: Vida media de la eliminación asociada con una pendiente terminal de una curva de concentración-tiempo sema-logarítmica;Vz: Volumen de distribución durante la fase terminal; Vss : Volumen de distribución en estado-estático; na no aplicable.

Reconocimientos

Los autores le agradecen a todo el personal involucrado en los estudios.

Aprobación ética

Para los dos estudios descritos subsecuentemente, los procedimientos en animales se condujeron en cumplimiento con la legislación nacional de bienestar animal y fueron aprobados pro comités relevantes de ética. El primer estudio (Estudio 1) fue conducido en Australia, aprobado por el Comité de Ética Animal en el sitio de la prueba y fue conducido con el permiso de la prueba otorgado por la Autoridad de Plaguicidas y Medicinas Veterinarias Australianas (APVMA). El segundo estudio (Estudio 2) fue conducido en Suiza, aprobado por el Oficial de Bienestar Animal de la compañía y conducido bajo un permiso de experimentación en animales de las autoridades cantonales suizas.

Consentimiento para publicación

No aplicable.

Disponibilidad de datos y material

No aplicable.

Intereses competentes

CET, WS y MJ son empleados de Elanco.

Fondos

Todos los estudios fueron patrocinados por Elanco.

Contribuciones de los autores

CET realizó un manuscrito preliminar y todos los autores revisaron y aprobaron la versión final. MJ fue autor de los diseños del estudio, WS llevó a cabo los análisis farmacocinéticos y todos los autores participaron en el resultado de la interpretación y la finalización del estudio.

Referencias

1. Rust MK. The biology, ecology and management of the cat flea. Annu Rev Entomol. 1997;452:451-73

2. Bowman, Dwight D. Georgis' parasitology for veterinarians. St Louis: Elsevier Health Sciences, 2014. p 55-63

3. Jongejan F, Uilenberg G. The global importance of ticks. Parasitology. 2004;129 Suppl:3–14.

4. Coles TB and Dryden MW. Insecticide/acaricide resistance in fleas and ticks infesting dogs and cats. Parasit Vectors. 2014;7:8.

5. Shoop WL, Hartline EJ, Gould BR, Waddell ME, McDowell RG, Kinney JB, et al. Discovery and mode of action of afoxolaner, a new isoxazoline parasiticide for dogs. Vet Parasitol. 2014;201:179-89.

6. McTier TL, Chubb N, Curtis MP, Hedges L, Inskeep GA, Knauer CS, et al. Discovery of sarolaner: a novel, orally administered, broad-spectrum, isoxazoline ectoparasiticide for dogs. Vet Parasitol. 2016;222:3-11.

7. Gassel M, Wolf C, Noack S, Williams H, Ilg T. The novel isoxazoline ectoparasiticide fluralaner: selective inhibition of arthropod γ-aminobutyric acid- and L-glutamate-gated chloride channels and insecticidal/acaricidal activity. Insect Biochem Mol Biol. 2014;45:111-24.

8. Letendre L, Huang R, Kvaternick V, Harriman J, Drag M, Soll M. The intravenous and oral pharmacokinetics of afoxolaner used as a monthly chewable antiparasitic for dogs. Vet Parasitol. 2014;201:190-7.

9. Ozoe Y, Asahi M, Ozoe F, Nakahira K, Mita T. The antiparasitic isoxazoline A1443 is a potent blocker of insect ligand-gated chloride channels. Biochem Biophys Res Commun. 2010;391:744-9.

10. Lahm GP, Cordova D, Barry JD, Pahutski TF, Smith BK, Long JK, et al. 4-Azolylphenyl isoxazoline insecticides acting at the GABA gated chloride channel. Bioorg Med Chem Lett. 2013;23:3001-6.

11. Kuntz E, Kammanadiminti S. Safety evaluation of lotilaner in dogs after oral administration as flavoured chewable tablets (Credelio™). Parasit Vectors. (in press).

12. Cavalleri D, Murphy M, Seewald W, Drake J, Nanchen S. Assessment of the speed of flea kill of lotilaner (Credelio™) throughout the month following oral administration to dogs. Parasit Vectors. (in press).

13. Cavalleri D,Murphy M, Seewald W, Drake J, Nanchen S. Assessment of the onset of lotilaner (Credelio™) speed of kill of fleas on dogs. Parasit Vectors. (in press).

14. Murphy M, Cavalleri D, Seewald W, Drake J, Nanchen S. Laboratory evaluation of the speed of kill of lotilaner (Credelio™) against *Ixodes ricinus* ticks on dogs. Parasit Vectors. (in press).

15. Karadzovska D, Chappell K, Coble S, Murphy M, Cavalleri D, Wiseman S, et al. A randomized, controlled field study to assess the efficacy and safety of lotilaner flavored chewable tablets (CredelioTM) in eliminating fleas in client-owned dogs in the USA. Parasit Vectors. 2017 (In press).

16. Cavalleri D, Murphy M, Seewald W, Drake J, Nanchen S. A randomized, blinded, controlled field study to assess the efficacy and safety of lotilaner tablets (Credelio^{TM}) in controlling fleas in client-owned dogs in Europe. Parasit Vectors. (in press).

17. FDA: Guidance for Industry, Bioanalytical Method Validation. Rockville: U.S. Department of Health and Human Services, Food and Drug Administration, Center for Drug Evaluation and Research (CDER), Center for Veterinary Medicine (CVM), 2001.

18. EMEA: Guideline on Bioanalytical Method validation, The European Agency for the Evaluation of Medicinal Products/Committee for Medicinal Products for Human Use, EMEA/CHMP/EWP/192217/2009, 2011.

19. Walther FM, Allan MJ, Roepke RKA, Nuernberger MC. The effect of food on the pharmacokinetics of oral fluralaner in dogs. Parasit Vectors. 2014;7:84.

20. Toutain PL, Bousquet-Mélou A: Bioavailability and its assessment. J Vet Pharmacol Ther 2004;27:455–66.

21. Toutain PL, Bousquet-Mélou A: Clearance. J Vet Pharmacol Ther 2004;27:415–25.

22. Toutain PL, Bousquet-Mélou A: Volumes of distribution. J Vet Pharmacol Ther 2004;27:441–53.

23. Toutain PL, Bousquet-Mélou A: Plasma terminal half life. J Vet Pharmacol Ther 2004;27:427–39.

24. Kilp S, Ramirez D, Allan MJ, Roepke RK, Nuernberger MC. Pharmacokinetics of fluralaner in dogs following a single oral or intravenous administration. Parasit Vectors. 2014;7:85.

Leyenda de las figuras

Fig. 1 Estructura molecular de lotilaner ((5-[5-(3,4,5-tricloro-fenil)-5-trifluorometil-4,5-dihidroisoxazol-3-il] -3-metil-tiofeno-2-ácido carboxílico [(2,2,2-trifluoro-etilcarbamoil)-metil]-amida)

Fig. 2 Gráficas del perfil medio de la dosis-normalizada (a 15 mg/kg) (± error estándar) de lotilaner después de la administración oral bajo diferentes regímenes de alimentación

Fig. 3 Gráficas del perfil medio de la dosis-normalizada (a 1 mg/kg) (± error estándar) de lotilaner después de la administración intravenosa u oral a perros alimentados o ayunados

Tabla 1 Efecto de diferentes regímenes de alimentación sobre la biodisponibilidad: comparaciones estadísticas (Estudio 1)

**Grupos de tratamiento**		**Proporción**[a] **(primero/segundo)**	**90% CI**	**Valor-*t***	**Valor-*P***[b]
**Primero**	**Segundo**				
Alimentado 30 min antes	Alimentado al dosificar	1.137	0.820–1.576	$t_{(20)} = 0.68$	0.5052
Alimentado 30 min antes	Alimentado 1/3 de ración	1.252	0.904–1.735	$t_{(20)} = 1.19$	0.2484
Alimentado 30 min antes	Alimentado 30 min después	1.029	0.743–1.426	$t_{(20)} = 0.15$	0.8799
Alimentado 30 min antes	Ayunado	2.250	1.624–3.118	$t_{(20)} = 4.29$	**0.0004**
Alimentado al dosificar	Alimentado 1/3 de ración	1.101	0.795–1.526	$t_{(20)} = 0.51$	0.6154
Alimentado al dosificar	Alimentado 30 min después	0.905	0.653–1.255	$t_{(20)} = -0.53$	0.6050
Alimentado al dosificar	Ayunado	1.979	1.428–2.742	$t_{(20)} = 3.61$	**0.0018**
Alimentado 1/3 de ración	Alimentado 30 min después	0.822	0.593–1.139	$t_{(20)} = -1.04$	0.3126
Alimentado 1/3 de ración	Ayunado	1.797	1.297–2.490	$t_{(20)} = 3.10$	**0.0057**
Alimentado 30 min después	Ayunado	2.186	1.577–3.029	$t_{(20)} = 4.13$	**0.0005**

[a]Proporción de biodisponibilidades i.e. proporción de $AUC_{última}$

[b]Los valores-*P* en negrita denotan significancia

Tabla 2 Media ± desviación estándar de los parámetros farmacocinéticos de lotilaner en perros adultos Beagle después de una sola administración oral con la dosis blanco de 20 mg/kg a perros ayunados, oralmente a perros alimentados, o con la dosis blanco de 3.0 mg/kg intravenosamente. Todos los valores (Media y desviación estándar) están basados en estadísticas geométricas resumidas (correspondientes a las estadísticas resumidas de los valores logarítmicos tranformados y luego vueltos a tranformar), excepto por T_{max} que se basa en la mediana

**Parámetros**	**Intravenoso, 3 mg/kg (*n* = 8)**	**Oral, alimentado 20 mg/kg (*n* = 12)**	**Oral, ayunado 20 mg/kg (*n* = 6)**
Dosis reales (mg/kg)	3.08-3.24	20.09-24.67	20.16-24.62
T_{max} (horas)	na	2 (range: 1–24)	4 (range: 2–24)
C_{max} (ng/ml)	na	4011 ± 990	1454 ± 849
$AUC_{última}$ (día*ng/ml)	10976 ± 1381	62,840 ± 17,346	18,592 ± 16,632
AUC_{inf} (día*ng/ml)	17844 ± 1937	118,600 ± 42,287	40,886 ± 31,915
C_{max} (dosis normalizada[a]) (ng/ml)	na	179.2 ± 43.5	65.4 ± 35.7
$AUC_{última}$ (dosis normalizada[a]) (día*ng/ml)	3436 ± 430	2806 ± 801	837 ± 731
AUC_{inf} (dosis normalizada[a]) (día*ng/ml)	5586 ± 666	5297 ± 1921	1840 ± 1406
$T_{1/2z}$ (día)	24.6 ± 5.9	30.7 ± 10.0	38.8 ± 11.2
MRT (día)	36.0 ± 8.5	45.3 ± 13.9	56.9 ± 14.8
CL (l/kg/día)	0.18 ± 0.02	na	na
V_z (l/kg)	6.35 ± 1.27	na	na
V_{ss} (l/kg)	6.45± 1.26	na	na
Biodisponibilidad (F%)	na	81.7	24.3

[a]Dosis-normalizada a 1 mg/kg

Tabla 3 Comparaciones entre grupos alimentados y ayunados después de la administración oral de lotilaner a 20mg/kg (Estudio 2).

**Parámetro**	**Proporción (ayunados a alimentados)**	**90% CI**	**Valor-t**	**Valor-*P*[a]**
$T_{1/2z}$	1.263	0.983–1.624	$t_{(23)} = -1.59$	0.1244
MRT	1.256	0.991–1.591	$t_{(23)} = -1.65$	0.1128
C_{max} (dosis normalizada)	0.365	0.265–0.502	$t_{(16)} = 5.51$	**< 0.0001**
$AUC_{última}$ (dosis normalizada)	0.298	0.201–0.441	$t_{(23)} = 5.29$	**< 0.0001**
AUC_{inf} (dosis normalizada)	0.347	0.238–0.507	$t_{(23)} = 4.80$	**< 0.0001**
Biodisponibilidad	0.298	0.201–0.441	$t_{(23)} = 5.29$	**< 0.0001**

[a]Los valores-*P* en negrita denotan significancia
