# Supplementary material for: The intravenous and oral pharmacokinetics of lotilaner in dogs
Source: Parasit Vectors. 2017 Nov 1;10:522. doi: 10.1186/s13071-017-2475-z (PMC5664907; doi:10.1186/s13071-017-2475-z)
Supplement: Supplementary file 2 — French translation of the Abstract. (PDF 17 kb) [file 13071_2017_2475_MOESM2_ESM.pdf]

Pharmacocinétique du lotilaner après administration par voie orale et intraveineuse chez le chien

Céline E. Toutain[1]*, Wolfgang Seewald[1] et Martin Jung[1]

[1]Elanco Santé Animale, Mattenstrasse 24a, CH-4058 Bâle, Suisse

*Correspondance : Céline E. Toutain
E-mails:
CET : celine.toutain@elanco.com
WS : wolfgang.seewald@elanco.com
MJ : martin.jung@elanco.com

Résumé

Introduction : Le lotilaner est un nouvel antiparasitaire oral de la classe des isoxazolines, développé chez le chien contre les infestations par les puces et les tiques. Il se présente sous forme de comprimés à mâcher aromatisés, formulés à partir d'énantiomère de configuration S pur (Credelio™). La pharmacocinétique du lotilaner a été établie chez le chien après administration par voie orale et intraveineuse et pour différents régimes alimentaires.

Méthodes : Vingt-six chiens adultes de race beagle ont été inclus dans une étude pharmacocinétique évaluant l'administration intraveineuse ou orale du lotilaner. Suite à l'administration orale de 20 mg/kg chez des animaux à jeun ou nourris, ou à l'administration intraveineuse de 3 mg/kg, des échantillons de sang ont été prélevés jusqu'à 35 jours après le traitement. Les effets de l'horaire des repas et de la quantité d'aliment ingérée avant ou après administration sur la biodisponibilité ont été évalués lors d'une étude distincte menée chez 25 chiens adultes. Les concentrations sanguines en lotilaner ont été mesurées par une méthode validée de chromatographie en phase liquide couplée à la spectrométrie de masse (LC-MS/MS). Les paramètres pharmacocinétiques ont été calculés par une analyse non compartimentale. De plus, la stabilité *in vivo* de l'énantiomère a été évaluée dans une étude analytique.

Résultats : Suite à l'administration par voie orale chez des animaux nourris, le lotilaner est rapidement absorbé, le pic de concentration sanguine étant atteint dans les 2 heures. La demi-vie est de 30.7 jours. La prise d'un repas favorise l'absorption et permet d'obtenir une biodisponibilité orale supérieure à 80 % tout en réduisant la variabilité inter-individuelle. De plus, le moment de la distribution du repas (30 minutes avant, pendant, ou 30 minutes après l'administration) ou la diminution de la ration alimentaire à un tiers de la ration journalière normale n'ont pas d'effet sur la biodisponibilité. Suite à son administration intraveineuse, le lotilaner a une clairance faible de 0.18 l/kg/jour, d'importants volumes de distribution Vz et Vss, respectivement de 6.35 et 6.45 l/kg, et une demi-vie de 24.6 jours. De plus, aucune racémisation du lotilaner n'est observée *in vivo*.
Conclusions : Les propriétés pharmacocinétiques du lotilaner, administré par voie orale sous forme de comprimé à mâcher aromatisé (Credelio™), ont fait l'objet d'une étude approfondie. Avec un Tmax de 2 heures et une demi-vie de 30.7 jours chez les chiens nourris, le lotilaner exerce un effet létal rapide sur les puces et les tiques et une efficacité prolongée pendant au moins un mois.
